# 本科仪器分析实验教学设计：响应面法优化QuEChERS结合气相色谱-质谱检测茶叶中联苯菊酯农药残留

**DOI:** 10.3724/SP.J.1123.2025.06006

**Published:** 2026-06-08

**Authors:** Chunxiu GU

**Affiliations:** 北京联合大学生物化学工程学院，北京 100023; Biochemical Engineering College，Beijing Union University，Beijing 100023，China

**Keywords:** 仪器分析实验, 样品前处理, 气相色谱-质谱, 联苯菊酯, 农药残留, 实验教学, instrumental analysis experiment, sample pretreatment, gas chromatography-mass spectrometry （GC-MS）, bifenthrin, pesticide residue, experimental teaching

## Abstract

本文针对制药工程专业本科实验教学需求，设计了一套基于QuEChERS-GC-MS联用技术检测茶叶中联苯菊酯农药残留的实验方案。实验内容包括样品前处理、QuEChERS实验条件的单因素筛选和响应面法优化、GC-MS分析方法建立、茶叶样品中联苯菊酯农药残留的测定以及实验数据处理等环节，旨在培养学生综合运用现代分析技术解决实际问题的能力。通过该实验，学生可以掌握GC-MS仪器设备的基本原理和操作技能，了解真实样品中农药残留分析的完整流程，体会样品前处理对复杂体系中痕量组分分析的重要性。在单因素实验选择的QuEChERS实验条件下，茶叶样品中联苯菊酯的回收率为85.9%；在响应面法优化的QuEChERS实验条件下，茶叶样品中联苯菊酯的回收率可达90.33%。在38份茶叶样品中，有12份样品检测出了联苯菊酯，最大检出值为0.911 mg/kg，均未超出食品安全国家标准GB 2763-2021规定的茶叶中联苯菊酯的最大残留限量。本教学设计包括实验前相关内容学习、实验中操作技能训练和实验后数据分析，不仅能激发学生的学习兴趣，还能培养学生严谨的科学思维，让学生在全流程学习过程中主动面对问题，积极分析问题，设法解决问题。

## Abstract (en)

This paper designs an experimental scheme to meet the undergraduate experimental teaching requirements of the Pharmaceutical Engineering major. The content focuses on the detection of bifenthrin pesticide residue in tea by QuEChERS-GC-MS. The experimental contents include sample pretreatment， single-factor screening and response surface methodology optimization of QuEChERS experimental conditions， establishment of a GC-MS analysis method， determination of bifenthrin pesticide residues in tea samples， and experimental data processing. It aims to cultivate students’ ability to comprehensively apply modern analytical techniques to solve practical problems. Through this experiment， students can master the basic principles and operational skills of GC-MS instruments and equipment， and understand the complete process of pesticide residue analysis in real samples， and recognize the importance of sample pretreatment for the analysis of trace components in complex systems. Under the QuEChERS experimental conditions screened in the single-factor experiment， the recovery rate of bifenthrin in the tea samples was 85.9%. Under the QuEChERS experimental conditions optimized by the response surface methodology， the recovery rate of bifenthrin in tea samples can reach 90.33%. Among the 38 tea samples， bifenthrin was detected in 12 samples， with the maximum detected value of 0.911 mg/kg. None of them exceeded the maximum residue limit of bifenthrin in tea stipulated in the National Food Safety Standard GB 2763-2021. This teaching design involves learning relevant content before the experiment， training operational skills during the experiment， and conducting data analysis after the experiment. It can not only stimulate students’ interest in learning， but also cultivate their rigorous scientific thinking. It enables students to proactively face problems， actively analyze them and try to solve them throughout the entire learning process.

仪器分析是重要的分析测试技术，熟悉并掌握各类仪器分析原理和实验技术是培养高素质工科人才的重要环节。仪器分析课程涉及大量抽象原理（如光谱、质谱、色谱、电化学等），通过实验教学，学生能直观理解仪器工作原理、操作流程及数据处理方法，巩固理论知识。传统的仪器分析实验教学多为验证性实验，很少涉及综合性实验案例^［1，2］^。为了培养学生的综合素质和创新思维，仪器分析实验设计应从授课对象的专业特点出发，与科研成果紧密结合。对于制药工程专业的学生来说，药食样品的检测能让学生接触真实应用场景，提升就业竞争力，缩短岗位适应期。

联苯菊酯是一种高效广谱拟除虫菊酯类杀虫剂，可用于防治棉铃虫、红铃虫、茶尺蠖、茶毛虫、菜青虫、小菜蛾、柑橘潜叶蛾等，在茶叶病虫害防治上被广泛使用^［3，4］^，因此也是茶叶中残留农药质量监控的重点对象。目前，茶叶中残留农药的检测主要有气相色谱（GC）技术^［5］^、高效液相色谱（HPLC）技术^［6］^，以及GC-MS^［7，8］^和HPLC-MS^［9，10］^等。GC-MS技术具有高灵敏度和高选择性，能够满足《中国药典》对农药残留检测的要求。茶叶中的农药残留含量极低且基质复杂，因此需进行适当的样品前处理以消除样品基底对分析结果的干扰和提高检测灵敏度。传统样品前处理技术，如索式提取法、液液萃取法、超声波萃取法、固相萃取法等虽已被用于农药残留的富集，但存在操作烦琐、溶剂消耗量大、分析时间长等缺点。近年来新兴的分散固相萃取净化技术QuEChERS具有简便、高效、低耗和环境友好等特点，符合制药行业绿色分析的发展趋势，已被广泛应用^［11-15］^。

本实验通过测定茶叶中痕量残留农药，将样品处理条件的优化引入本科教学，通过完整的分析流程设计，使学生系统掌握从样品前处理到仪器分析的全过程。与传统液液萃取相比，QuEChERS技术操作简便，有机溶剂用量少，更适合本科实验教学。同时，实验教学将理论知识与实际应用相结合，有助于培养学生解决实际问题的能力，为培养新时代符合国家和社会需求的高素质人才奠定基础。

## 1 实验部分

### 1.1 仪器、试剂与材料

7890A-5975C气相色谱-质谱联用仪（美国Agilent公司）；MS3涡旋振荡器（德国IKA公司）；N-EVAP-24氮吹仪（美国Organomation公司）；Allegra X-30R Centrifuge高速离心机（美国Beckman公司）；ME2002E电子天平（瑞士Mettler Toledo公司）。

联苯菊酯标准品（100 mg/L，农业部环境保护科研监测所）；*N*-丙基乙二胺（PSA）吸附剂和十八烷基硅烷（C18）吸附剂（美国Agilent公司）；乙醇、无水硫酸镁、乙腈、氯化钠、丙酮和乙酸乙酯（均为分析纯，北京惠宝化学试剂公司）；茶叶样品（北京超市购买）。

### 1.2 样品前处理

准确称取2.00 g粉碎并过40目筛的茶叶样品，置于50 mL具塞离心管中，加入5 mL饱和NaCl水溶液，静置15 min，然后加入15 mL乙腈，涡旋振荡10 min，加入298.58 mg无水硫酸镁，继续涡旋振荡10 min，4 500 r/min离心5 min。吸取12 mL上清液加到20 mL离心管中，加入90.97 mg PSA及51.07 mg C18，涡旋5 min，4 500 r/min离心5 min，准确吸取6 mL上清液于10 mL玻璃试管中，于45 ℃水浴氮气吹干，用1 mL乙酸乙酯-丙酮（1∶1，体积比）溶解残渣，过膜后待测。

空白茶叶样品制备：将茶叶样品置于烧杯中，加入75%乙醇溶液浸泡30 min后，洗净乙醇，沸水浸泡30 min，多次重复，直至样品检测不到联苯菊酯，烘干、粉碎，过40目筛备用。

### 1.3 仪器分析条件

GC条件：TG-5 MS石英毛细管柱（30 m×0.25 mm×0.25 μm）；进样口温度为280 ℃，载气为高纯氦气，流速1.00 mL/min；柱温起始为40 ℃，保持2 min，以30 ℃/min升至120 ℃，保持5 min，再以10 ℃/min升至240 ℃，保持8 min，最后以8 ℃/min升至300 ℃，保持1 min；不分流进样。

MS条件：离子源为EI源，离子源温度280 ℃，电子能量70 eV，接口温度280 ℃，碰撞气为氩气（99.999%），选择反应监测（SRM）模式，联苯菊酯保留时间25.36 min，定性离子对181.1>165.2（碰撞电压10 eV），定量离子对181.2>165.9（碰撞电压24 eV）。

## 2 结果与讨论

### 2.1 QuEChERS条件的选择和优化

QuEChERS是一种用于复杂样品的前处理技术。其实施步骤可以简单归纳如下：样品粉碎、乙腈提取、盐析剂除水、吸附剂除杂，然后保留上清液待测。其中，盐析剂和吸附剂的选择对目标物的萃取至关重要^［16，17］^。

#### 2.1.1 盐析剂和吸附剂的选择

NaCl、无水Na_2_SO_4_和无水MgSO_4_是QuEChERS中最常用的盐析剂。盐析剂能促进待测物向提取剂分配，以提高目标化合物的回收率。刘佳等^［18］^选择无水Na_2_SO_4_+无水MgSO_4_+NaCl盐析剂组合测定茶叶中拟除虫菊酯类农药残留；张婧文等^［19］^考察了无水Na_2_SO_4_+NaCl和无水MgSO_4_+NaCl两种盐析剂组合对新烟碱类杀虫剂回收率的影响，认为无水Na_2_SO_4_+NaCl的整体回收率更高；潘思竹等^［20］^选择无水MgSO_4_+NaCl盐析剂组合测定茶叶中联苯菊酯残留；杨延等^［21］^也选择无水MgSO_4_+NaCl盐析剂组合测定茶叶中农药残留。我们的初步研究发现无水MgSO_4_+NaCl盐析剂组合对联苯菊酯的分离更有利。

PSA、C18和GCB是QuEChERS常使用的吸附剂。PSA能够有效去除脂肪酸、糖类和色素等极性干扰，而C18对非极性物质的吸附能力较强，GCB常用于吸附色素。我们分别研究了3种吸附剂的净化效果，发现GCB在实验中的净化作用有限，PSA和C18都有很好的净化效果。因此，选择PSA和C18作为净化阶段的吸附剂。

#### 2.1.2 单因素条件优化

以联苯菊酯回收率为考察指标，分别研究了无水MgSO_4_、PSA和C18的用量对目标物萃取的影响，结果如图1所示。当无水MgSO_4_、PSA和C18的用量分别为250、100、50 mg时，联苯菊酯的回收率可达到最大值（约为85.9%）。

**图1:**
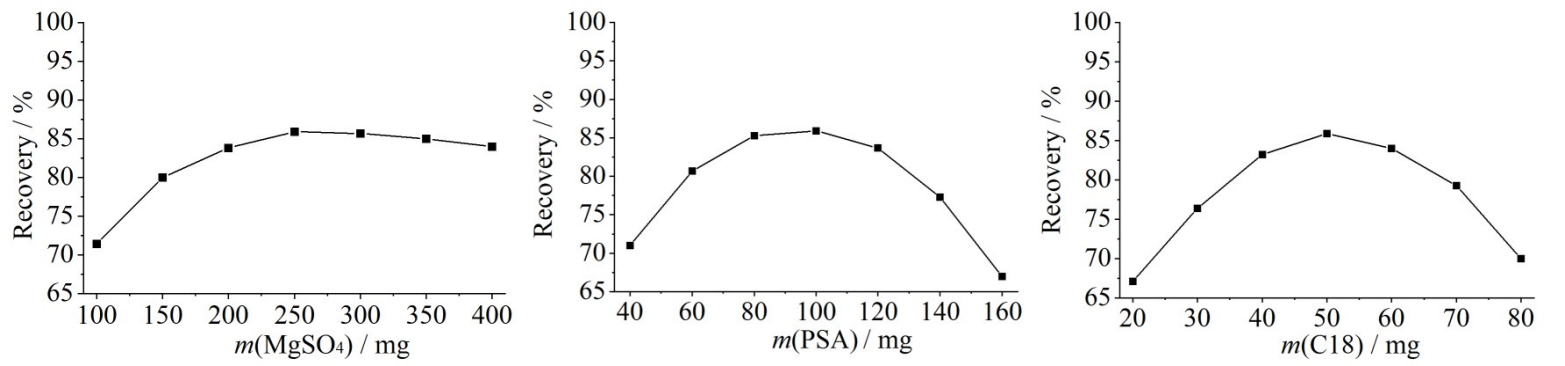
无水MgSO_4_、PSA和C18的用量对联苯菊酯回收率的影响

#### 2.1.3 响应面试验

响应面法（response surface methodology， RSM）是一种研究多因素对响应指标影响的优化技术，它通过考察不同因素之间的交互作用来综合优化不同因素对响应指标的影响^［22，23］^。在单因素实验结果的基础上，以筛选值为中心点，根据Box-Benhnken实验设计原理，以无水MgSO_4_、PSA和C18用量为自变量，以联苯菊酯的回收率为响应指标，设计的三因素三水平见表1，17个响应面设计试验和结果见表2。

**表1:** Box-Behnken实验设计的因素和水平

Level	*A*（*m*（MgSO_4_））/mg	*B*（*m*（PSA））/mg	*C*（*m*（C18））/mg
‒1	200	80	40
0	250	100	50
1	300	120	60

**表2:** 响应面设计和试验结果

Trial	*A*	*B*	*C*	Recovery/%
1	300	80	50	85.2
2	250	100	50	85.9
3	200	100	60	75.9
4	200	100	40	72.3
5	300	100	40	82.5
6	250	100	50	85.9
7	250	120	40	65.3
8	250	100	50	85.9
9	200	120	50	80.5
10	300	100	60	83.5
11	250	100	50	85.9
12	250	120	60	71.7
13	200	80	50	78.3
14	300	120	50	81.4
15	250	100	50	85.8
16	250	80	40	75.3
17	250	80	60	82.1

采用Design Expert软件进行三因素回归拟合分析。无水MgSO_4_用量（*A*）、PSA用量（*B*）和C18用量（*C*）对联苯菊酯的回收率（*Y*）影响的回归方程如下：

*Y*=85.88+3.2*A*-2.75*B*+2.23*C*-1.5*AB*-0.65*AC*-0.1*BC*+0.21*A*^2^-4.74*B*^2^-7.54*C*^2^

在所选因素的水平范围内，对结果的影响顺序如下：MgSO_4_用量>PSA用量>C18用量。绘制的三维曲面图和等高线图见图2~4。

**图2:**
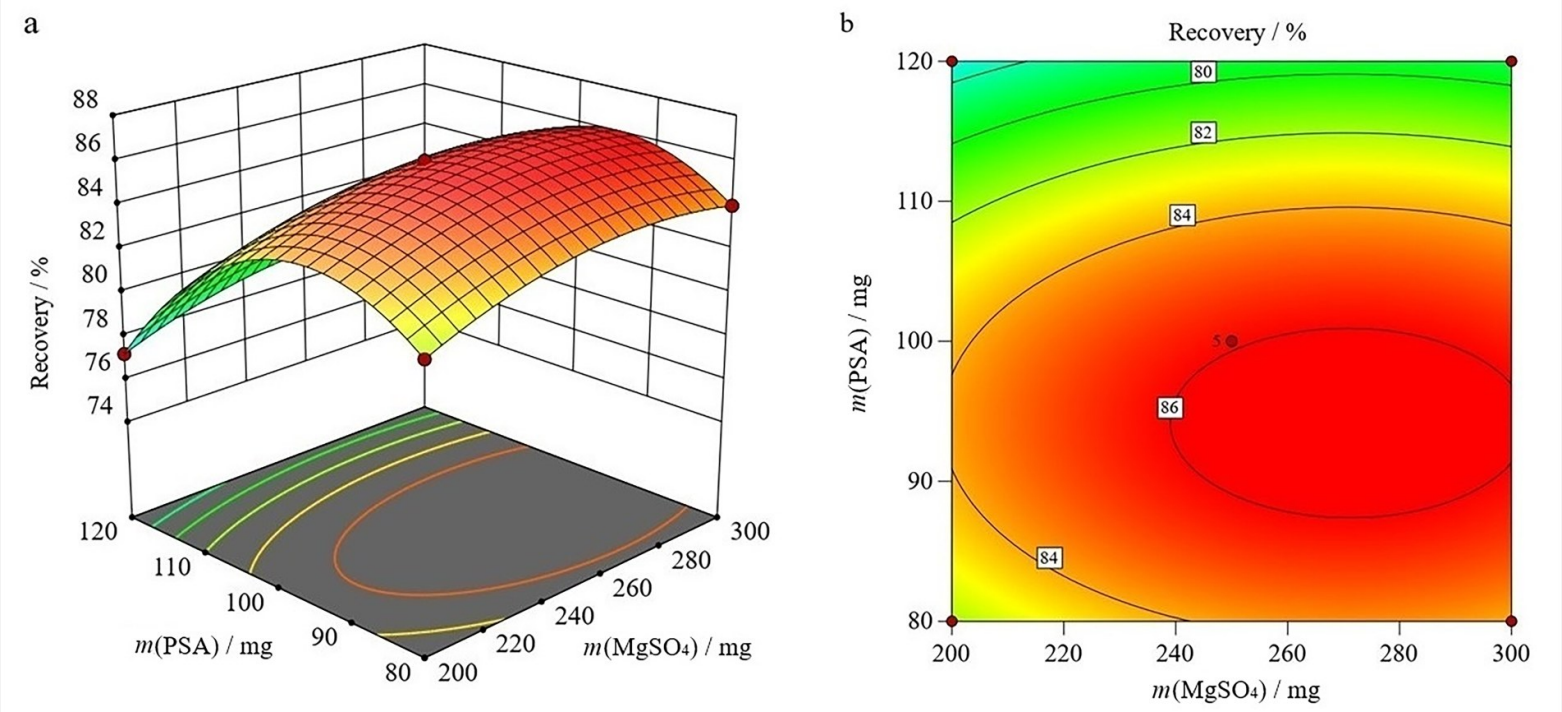
无水MgSO_4_用量和PSA用量的交互作用对联苯菊酯回收率影响的（a）三维曲面图和（b）等高线图

**图3:**
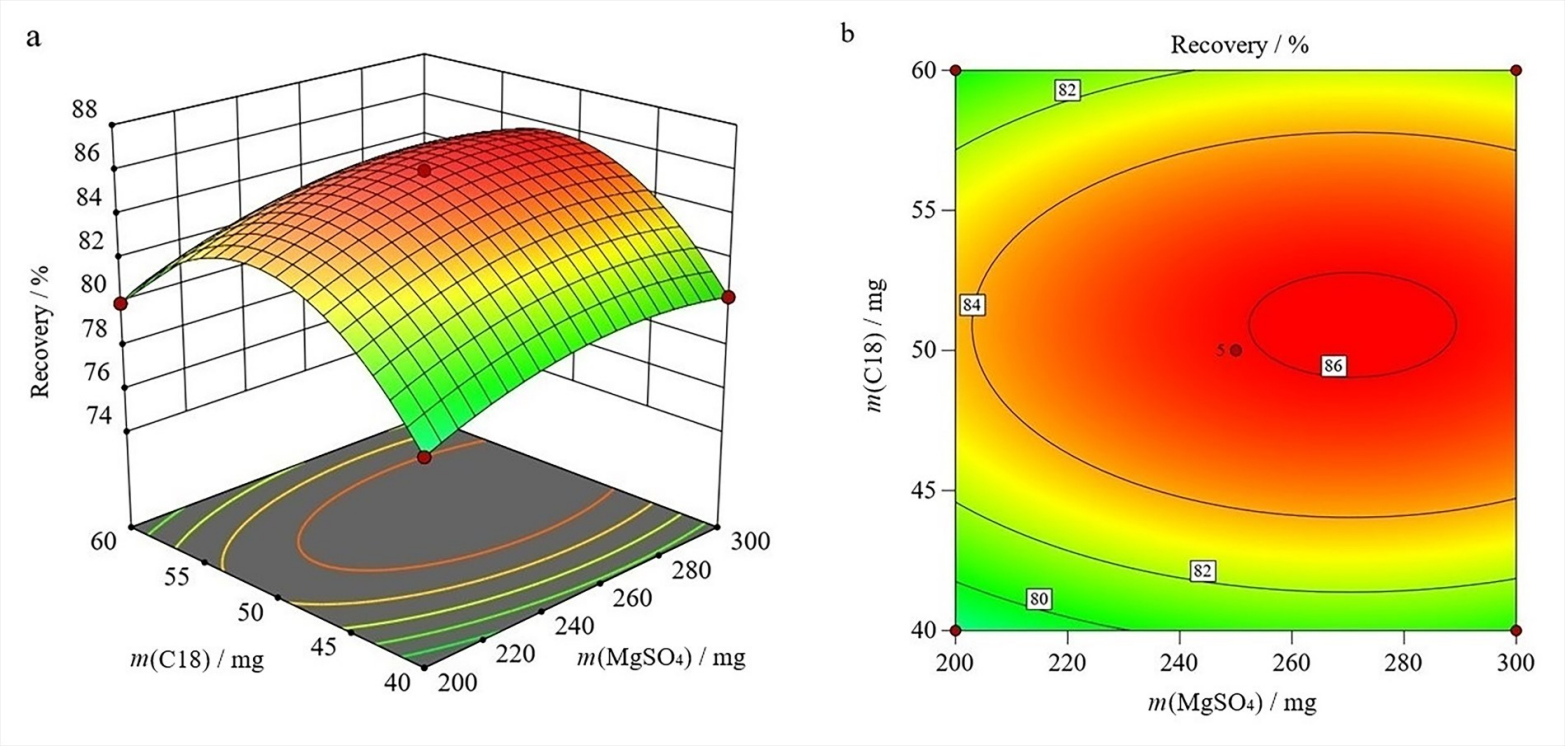
无水MgSO_4_用量和C18用量的交互作用对联苯菊酯回收率影响的（a）三维曲面图和（b）等高线图

**图4:**
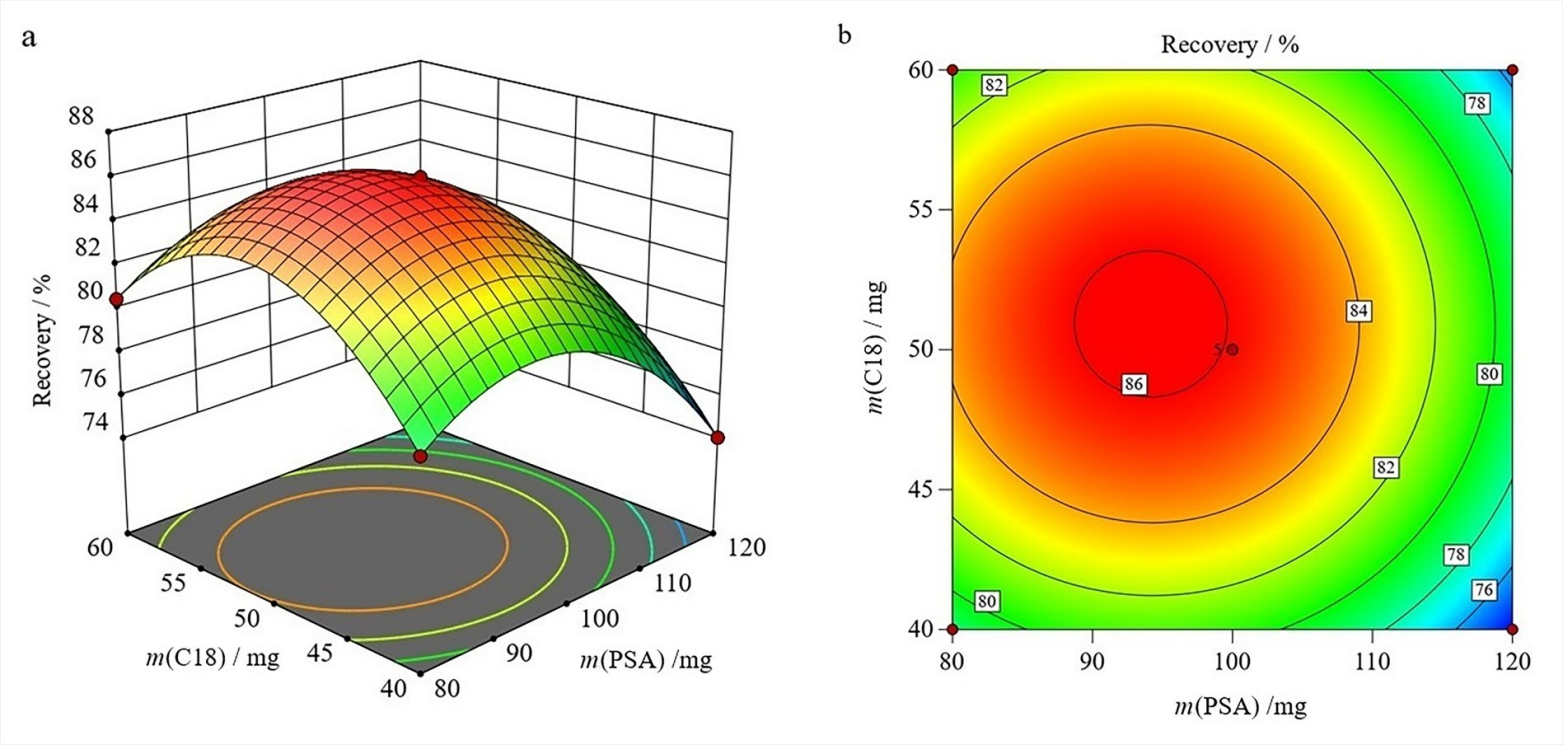
PSA用量和C18用量的交互作用对联苯菊酯回收率影响的（a）三维曲面图和（b）等高线图

如图2a所示，随着无水MgSO_4_用量和PSA用量的增加，联苯菊酯回收率也逐渐提高。当无水MgSO_4_用量达到298.58 mg和PSA用量达到90.97 mg时，联苯菊酯能获得相对高的回收率。其等高线图（图2b）呈椭圆形状，表明这两个因素的交互作用很明显。

如图3a所示，当无水MgSO_4_用量达到298.58 mg和C18用量达到51.07 mg时，联苯菊酯能获得相对高的回收率。从其等高线图（图3b）可以看到这两个因素的交互作用也很明显。

如图4a所示，当PSA用量达到90.97 mg和C18用量达到51.07 mg时，联苯菊酯能获得相对高的回收率。其等高线图4b几乎呈圆形，表明这两个因素的交互作用很弱。

综上所述，响应面优化的各试剂用量如下：298.58 mg无水MgSO_4_、90.97 mg PSA和51.07 mg C18，此时预测的联苯菊酯回收率为90.33%。响应面优化结果与单因素筛选的条件相比，联苯菊酯回收率有所提高，这对痕量组分的测定非常重要。

### 2.2 方法学考察

用空白茶叶样品按照1.2节方法制备足量的空白底液，并确认在空白底液中检测不到联苯菊酯。用空白底液配制一系列不同质量浓度的联苯菊酯标准溶液，利用GC-MS测定联苯菊酯的含量。结果表明，峰面积的响应值（*Y*）与联苯菊酯的浓度（*X*，μg/L）呈现出很好的线性关系*Y*=4.38×10^4^ *X*+63.7（*R*^2^=0.999 3），线性范围为1.0~800.0 μg/L；通过3倍信噪比和10倍信噪比可分别得到检出限（LOD）为0.06 μg/L和定量限（LOQ）为0.09 μg/L。按照1.2节的样品处理方法，通过样品量换算可得出茶叶样品中联苯菊酯的检出限为1.0×10^-4^ mg/kg，定量限为1.5×10^-4^ mg/kg。在线性范围内，配制10个标准样品，对每个标准样品进行8次重复测定，得到联苯菊酯的回收率为94.6%~105.3%，相对标准偏差（RSD）为1.3%~2.6%。

### 2.3 实际样品分析

用本方法测定了38份茶叶样品，结果显示有12份样品检测出联苯菊酯，如表3所示，检出率达31.6%，最大检出值为0.911 mg/kg，均未超出食品安全国家标准GB 2763-2021规定茶叶中联苯菊酯最大残留限量（5 mg/kg）。

**表3:** 市售茶叶样品的检测结果

Sample	Detection value/（mg/kg）
Sample 3	0.085
Sample 7	0.267
Sample 9	0.173
Sample 11	0.059
Sample 12	0.019
Sample 15	0.022
Sample 18	0.487
Sample 21	0.911
Sample 25	0.386
Sample 28	0.008
Sample 29	0.635
Sample 34	0.195

## 3 实验的组织实施及教学安排

本实验为制药工程专业仪器分析实验课程的开放型实验项目，要求学生：“用响应面法优化QuEChERS萃取净化茶叶的实验条件，并用GC-MS检测提取液中联苯菊酯含量”。学生3~4人为一个实验小组，共同查阅相关文献资料、研讨设计实验方案、协作完成实验内容并提交实验报告。该实验分为3个阶段，按每周5天、每天8学时，在5周内完成。如果将第一阶段和第三阶段安排在课外，则可以只集中两周实验教学，具体教学内容安排如表4所示。

**表4:** 仪器分析实验教学内容安排

Stage	Content	Class hour/h
Study relevant theories and literature	residual pesticides in medicinal and food materials	8
	separation and analysis methods for residual pesticides	8
	sample pretreatment techniques and principles	12
	optimization method of experimental conditions	4
	learn the Design Expert software	8
	GC separation theory and MS analysis theory	8
	GC-MS operation practice	16
	experimental scheme design	16
Master operational skills	prepare various solutions	8
	preparation of tea samples and blank samples	16
	single-factor experiment of QuEChERS extraction	8
	response surface optimization series tests	16
	experimental conditions for GC and MS	8
	method for the determination of bifenthrin by GC-MS	8
	determination of real samples	16
Sort out the data and write the experimental report	analysis of single-factor extraction conditions	4
	optimization of the response surface of extraction conditions	12
	establishment of the GC-MS method	4
	analysis of tea samples	8
	write a comprehensive experimental report	12

第一阶段约2周，学生通过阅读相关的文献资料，了解食品和药品中残留农药的测定方法，学习QuEChERS原理和实施方法，学会响应面法优化方法和Design Expert软件使用方法，熟悉GC-MS仪器设备结构并掌握其规范操作方法；在此基础上，完成包含具体数据的详细实验方案，并准备相关实验试剂和材料。

第二阶段约2周，完成全部实验内容，包括：各种溶液的配制，茶叶样品和空白样品的准备，QuEChERS萃取净化茶叶中联苯菊酯不同实验条件的单因素筛选实验和响应面法优化试验，建立GC-MS测定溶液中联苯菊酯方法的系列实验，完成茶叶样品中联苯菊酯的测定。

第三阶段约1周，完成实验数据的整理、分析和相关计算，每个实验小组撰写并提交一份完整的实验报告。

## 4 教学反思及注意事项

在第一阶段，教师需引导学生查阅与实验密切相关的文献资料，包括药材药品中残留农药的测定方法、样品前处理技术等，让学生快速聚焦本实验的重点与难点：茶叶中联苯菊酯测定方法和QuEChERS萃取净化实验条件的选择。对每个小组设计的实验方案，教师要仔细把关，以确保实验方案的可行性。

在第二阶段，教师需及时指导学生分析每一步的实验数据，评估实验结果是否符合预期。对满意的实验结果要总结实验过程的关键步骤，对不满意的实验结果要分析可能的原因以及是否需要补做实验。通过反复多次的实验和分析，培养学生严谨踏实的工作作风。每个学生小组在实验初期往往会对实验投入很大的时间和热情，学生在这个阶段会提出很多问题，指导教师要随时解答学生的各种问题。

QuEChERS实验条件的选择和优化是本实验开放性较大的内容，学生可以调整和选择各种不同的实验条件，如可以选用无水Na_2_SO_4_和GCB等，也可以把它们选作优化条件等。但为了保证能在规定时间内完成任务，尽量选择三因素三水平的优化设计，最多允许个别小组尝试四因素三水平的响应面试验（这需要完成29组试验）。

在样品准备阶段，让每个实验小组采购并准备一份合格的茶叶样品，最终各小组要完成全部样品的测定。实验过程中，很多小组除了采购的茶叶样品，还有学生从家里带来了茶叶样品，这些学生对自己的样品在各个小组的测定结果尤为关注。

实验过程中需让学生时刻牢记安全第一，包括开门关门、开机关机以及废物处理等所有细节；GC-MS需要在培训熟练并考核合格后才能独立操作使用；在实验过程的任何环节遇到偶发情况时，都要第一时间找指导教师处理。

## 5 结语

本实验是在制药工程专业的仪器分析实验项目上进行的扩展，增加了先进的样品前处理技术和多因素实验条件的优化方法。实验过程涉及一系列基础实验操作，可以锻炼学生的动手能力。GC-MS的认识和使用是这个实验的教学重点，学生在教师指导下需要反复多次完成GC-MS的调试和应用，最终学生都能够达到熟练操作的程度，这在一定程度上增强了学生使用大型分析仪器的信心。该实验不同于传统的验证实验模式，自始至终都以学生作为分析问题和解决问题的主体，有利于学生科学思维的养成和综合能力的培养。
